# The Maryland Dental Bill

**Published:** 1884-03

**Authors:** 


THE MARYLAND DENTAL BILL.—The following Bill to Regulate the Practice of Dentistry in the State of Maryland, has passed the Senate and House of Delegates.

Section 1. *Be it enacted by the General Assembly of Maryland*, That it shall be unlawful for any person who is not, at the time of the passage of this Act, engaged in the practice of dentistry in this State, to practice dentistry, unless he or she shall have obtained a certificate as hereinafter provided, or shall hold a diploma from a University or College chartered by or under the laws of this State, authorized to grant diplomas in Dental Surgery.

Sec. 2. *Be it enacted*, That a Board of Examiners, to consist of five reputable practicing dentists, is hereby created, whose duty it shall be to carry out the purposes and enforce the provisions of this Act, the members of said board shall be appointed by the Governor, who shall select them from the dentists residing in this State; *provided*, that none of said board shall be pecuniarily connected with any Dental College or dental department of any College or University: the term for which the members of said board shall hold their offices, shall be for four years, except that two members of the board, first to be appointed under this Act, shall be designated by the Governor to hold their offices for the term of two, and three for four years, respectively, unless sooner removed by the Governor, and until their successors shall be duly appointed; in case of a vacancy occurring in said board, such vacancy shall be filled in like manner by the Governor.

Sec. 3. *Be it enacted*, That said board shall choose one of its members President and one Secretary thereof, it shall fix the time and place of its meeting or meetings, a majority of said board shall, at all times constitute a quorum, and the proceedings thereof shall, at all reasonable times, be open to public inspection; the board shall also make an annual report of its proceedings to the Governor.

Sec. 4. *Be it enacted*, That within six months from the time this Act, takes effect, it shall be the duty of every person who is at that time engaged in the practice of dentistry in this State, to cause his or her name and residence or place of business to be registered with said Board of Examiners, who shall keep a book for that purpose; the statement of every such person shall be verified under oath before a Notary Public or Justice of the Peace, in such a manner as may be prescribed by the said Board of Examiners; every person who shall so register with said board, as a practitioner of dentistry, may continue to practice the same as such and shall receive a certificate of such registration upon his or her paying the said board one dollar for such certificate.

Sec. 5. *Be it enacted*, That any and all persons who shall desire to commence such practice may appear before said board at any of its regular meetings, and be examined with reference to their knowledge and skill in Dental Surgery, and if the examination of any such person or persons shall prove satisfactory to said board, the Board of Examiners shall issue to such persons, as they shall find to possess the requisite qualifications, a certificate to that effect, in accordance with the provisions of this Act, upon the payment of one dollar for such certificate; all certificates issued by said board shall be signed by its officers, and such certificates and diplomas granted as aforesaid, shall be *prima facie* evidence of the right of the holder to practice dentistry in the State of Maryland.

Sec. 6. *Be it enacted*, That any person who shall wilfully violate any of the provisions of this Act, shall be deemed guilty of a misdemeanor, and upon conviction thereof in any court having criminal jurisdiction, may be fined not less than fifty dollars nor more than three hundred dollars, or be confined not more than six months in the county jail, in the discretion of the court; all fines received under this Act, shall be paid into the common school fund of the city or county in which such conviction takes place.

Sec. 7. *Be it enacted,* That one member ot said board may grant any certificate provided for in this Act, to any applicant, upon presentation by such applicant of the evidence requisite for the obtaining said certificate which certificate shall remain in force until the next regular meeting of the said board after the granting of said certificate, and no longer, but no such certificate shall be issued by such member after such applicant has been rejected by said board.

Sec. 8. *Be it enacted,* That nothing in this Act shall be so construed as to interfere with the rights and privileges of physicians and surgeons in the discharge of their professional duties.

---

